# Study on Concave Direction Impact Performance of Similar Concave Hexagon Honeycomb Structure

**DOI:** 10.3390/ma16083262

**Published:** 2023-04-21

**Authors:** Guanxiao Zhao, Tao Fu, Jiaxing Li

**Affiliations:** 1 Faculty of Mechanical and Electrical Engineering, Kunming University of Science and Technology, Kunming 650093, China; zhaoguanxiao1123@163.com; 2 College of Mechanical and Electronic Engineering, Tarim University, Alar 843300, China

**Keywords:** honeycomb structure, similar concave hexagon, concave direction impact, in-plane impact, specific energy absorption, stress plateau

## Abstract

Based on the traditional concave hexagonal honeycomb structure, three kinds of concave hexagonal honeycomb structures were compared. The relative densities of traditional concave hexagonal honeycomb structures and three other classes of concave hexagonal honeycomb structures were derived using the geometric structure. The impact critical velocity of the structures was derived by using the 1-D impact theory. The in-plane impact characteristics and deformation modes of three kinds of similar concave hexagonal honeycomb structures in the concave direction at low, medium, and high velocity were analyzed using the finite element software ABAQUS. The results showed that the honeycomb structure of the cells of the three types undergoes two stages: concave hexagons and parallel quadrilaterals, at low velocity. For this reason, there are two stress platforms in the process of strain. With the increase in the velocity, the joints and middle of some cells form a glue-linked structure due to inertia. No excessive parallelogram structure appears, resulting in the blurring or even disappearance of the second stress platform. Finally, effects of different structural parameters on the plateau stress and energy absorption of structures similar to concave hexagons were obtained during low impact. The results provide a powerful reference for the negative Poisson’s ratio honeycomb structure under multi-directional impact.

## 1. Introduction

The effect of a negative Poisson’s ratio was first discovered in nature. The properties are opposite to those of common materials when stretched or compressed. Material with anomalous properties is called an auxetic material. Auxetic materials have excellent shear resistance, impact resistance, fracture resistance, energy absorption, and vibration isolation properties, etc. In 1987, Lakes made polyurethane foams with a negative Poisson’s ratio property [1]. Research on artificial auxetic materials began slowly from this point. Artificial auxetic materials are porous materials composed of tiny unit structures. The common microcell structures that have been made by researchers with negative Poisson’s ratio properties include: rotating rigid body structure [2], chiral structure [3], perforated plate structure [4], arrow structure [5], concave hexagonal structure [6], etc. Negative Poisson’s ratio materials have broad application prospects in engineering construction [7], biomedicine [8], aerospace [9], textiles [10], and navigation [11].

The honeycomb structure is the best topological structure covering the two-dimensional plane. It is a bionic structure. Compared with the common two-dimensional structure, the honeycomb structure has fewer consumables, a high strength-to-weight ratio, a high rigidity-to-weight ratio, high structural stability, good anti-fatigue characteristics, etc. In addition, the honeycomb structure also has an excellent energy absorption capacity [12], sound absorption capacity [13], heat absorption capacity [14], etc. Therefore, this kind of structure is widely used in automotive engineering, mechanical engineering, architecture, medicine, and biological engineering.

Auxetic materials and honeycomb structures can be well combined. The excellent mechanical properties of negative Poisson’s ratio honeycomb structures can be applied in everyday life. Therefore, negative Poisson ratio honeycomb materials have been extensively researched. Strek et al. [15] studied the influence of contact between half-cylinder elastic and plates. The contact pressure and length of the contact boundary when the half-cylinder elastic acts on the clamped auxetic plate and the plate covered with an auxetic layer were analyzed. When the Poisson’s ratio of the homogeneous auxetic plate is smaller, the contact pressure value will be larger, and length of the contact boundary will decrease. These two variables are closely related to Poisson’s ratio. The contact pressure and length of the contact boundary of the composite plate are closely related to the thickness of the auxetic layer. The clamped auxetic plate performs better than a plate covered with an auxetic layer. The significant influence of Poisson’s ratio on contact pressure (indentation issue) is clear, especially for extremely negative values close to minus 1. Mrozek et al. [16] studied a new type of deformed rotating block structure with holes and adjusted the structure’s geometric parameters to adjust the structure’s Poisson’s ratio (1 to −1). The dynamic mechanical properties (mechanical impedance, vibration transfer loss, transmissibility) of the structure are closely related to Poisson’s ratio. The dynamic mechanical properties of different structural parameters are analyzed in detail to determine the appropriate mechanical impedance and transmissibility. Novak et al. [17] made chiral auxetic cellular structures with alloy as the base material and determined the Poisson’s ratio of the structures in a variety of different structural parameters through experiments and simulations. The blast resistance performance of sandwich plates with chiral auxetic cellular structures under an explosion load is studied in detail. Compared with the positive Poisson’s ratio of the sandwich plate and homogeneous plate, the sandwich plate with negative Poisson’s ratio has better blast resistance performance. The research on the blast resistance performance of auxetic sandwich structures provides a great influence for the research on the blast resistance performance of composite sandwich structures. Alderson et al. [18] controls the Poisson’s ratio and the hole gradient of the foam by controlling the opening condition of the foam by technological means. The Poisson’s ratio of the foam is controlled from −1 to 1 and the effects of foam compression under different Poisson’s ratios were compared. This kind of auxetic foam research can be used in sports protective equipment, medical prosthetics, shoes, etc. Imbalzano et al. [19] introduced a sandwich plate consisting of an auxetic honeycomb structure and a metal section. They studied the mechanical properties of the structure under a pulse load and concluded that the pulse energy absorption of the sandwich plate was twice that of the solid plate when it was subjected to explosion impact, and the maximum displacement after impact was significantly reduced. Sarvestani et al. [20] proposed a semi-analytical method for structural and low-speed impact based on the modified high-order shear deformation theory and predicted the mechanical properties of sandwich structures by finite element analysis. The team also 3D-printed three different topological types of honeycomb sandwich plates and tested the samples for low-speed impact. It is concluded that the sandwich plate with a concave hexagonal honeycomb core has higher energy absorption capacity than that with square sandwich core and regular hexagonal sandwich core. Imbalzano et al. [21] studied the anti-violent performance of tensile honeycomb and ordinary honeycomb and used the Johnson–Cook model to describe the dynamic response of composite sandwiches under a high strain rate load. By changing the structural parameters of honeycomb, the performance of sandwiches with different structural parameters under the explosion load was studied and analyzed. Compared with the traditional sandwich plate, the auxetic honeycomb sandwich plate will have the effect of local stiffness enhancement under the explosion load.

In addition, the in-plane dynamic mechanical properties of auxetic honeycomb structures have also been extensively studied. Through experimental and numerical analysis, Günaydın et al. [22] compared the mechanical properties of 3D-printed back chiral quad ligamentous honeycomb and concave hexagonal honeycomb under in-plane compression. They concluded that the mechanical properties of in-plane back chiral quad ligamentous honeycomb were superior to concave hexagonal honeycomb, and the experimental results were verified. Liu et al. [23] analyzed the impact response and energy absorption characteristics of the concave honeycomb material under different strain rates, defined the irregular structure of the concave hexagonal honeycomb, and pointed out that the structural irregularity would enhance the energy absorption ability of the concave honeycomb under low velocity impact. Alomarah et al. [24] proposed a new structure with a negative Poisson’s ratio (re-entrant chiral auxetic, RCA) by combining the concave hexagon cell structure with the chiral structure. Later, a cylindrical geometric structure was added to the RCA, and its in-plane mechanical properties were improved. The Poisson’s ratio of honeycomb can be changed by adjusting the new cylindrical structure. Lu et al. [25] established the models of two honeycomb structures, the RP-H-RP and the H-RP, based on the regular hexagonal honeycomb structure. They analyzed the mechanical properties of the two models when both directions in the plane were subjected to explosive loads. They also summarized the deformation modes and energy absorption distribution of aluminum honeycomb under two kinds of blast types: direct explosion and indirect explosion. Alderson et al. [26] proposed the chiral structure of the concave triligament for the first time, compared four types of triligament honeycomb structures, and predicted the in-plane elastic constants and out-of-plane bending responses of these honeycomb structures. The results show that all four types of triligament honeycomb structures exhibit a negative Poisson’s ratio phenomenon when the load is placed in the plane. When the load is placed in the horizontal plane, the conventional chiral and back chiral honeycomb structures have the same positive Poisson’s ratio phenomenon as the regular hexagonal honeycomb structure, while the concave chiral and back chiral honeycomb structures have the same negative Poisson’s ratio phenomenon as the concave hexagonal honeycomb structure. Li et al. [27], through the analysis of explicit dynamics, studied the compression characteristics of uniaxial and biaxial in the honeycomb plane of three types of honeycombs: regular hexagonal honeycomb, concave hexagonal honeycomb, and mixed honeycomb. The deformation modes of three types under different impact modes are summarized and compared, and the energy absorption of the three honeycomb types under impact is analyzed and compared.

At present, a large number of researchers have extensively studied the in-plane characteristics of honeycomb structures with different element types, but the impact direction of the research is relatively singular. Among many negative Poisson’s ratio cell structures, the concave hexagon structure is special, and it will have different deformation modes and mechanical properties when impacted in different directions. Based on the traditional Concave hexagonal structure, we propose two types of similar concave hexagon honeycomb structures with a negative Poisson’s ratio. The relative density and critical velocity of the hexagonal structure are derived, and the dynamic impact response and energy absorption characteristics of each structure are analyzed by finite element simulation. This research reveals the double shape transformation of the concave direction of the similar concave hexagon structure when it is subjected to an in-plane impact. The research also provides basic information for the study of the impact performance of negative Poisson’s ratio honeycomb structures in multiple directions and applications in multiple scenarios.

## 2. Model Analysis

### 2.1. Analysis of Geometric Structure

**Figure 1:**
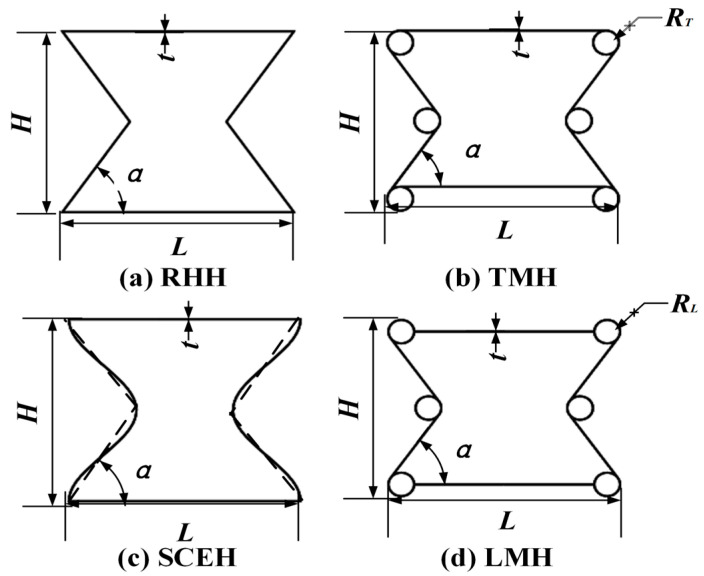
Diagrammatic sketch of similar concave hexagons. (**a**) Traditional concave hexagonal structure; (**b**) the top and bottom two ligaments are tangent to the ring, and the middle ring is tangent to the four adjacent ligaments; (**c**) the ligaments on both sides conform to a sinusoidal curve, where one period of the curve is T = H, and this structure is derived from RHH; (**d**) two ligaments at the top and bottom are connected to the ring, and the middle ring is tangent to the four adjacent ligaments.

The concave hexagon structure is derived from the traditional concave hexagonal structure (re-entrant hexagon honeycomb, RHH) [6]. In this paper, three kinds of honeycomb structures with concave hexagons are introduced which are different from the traditional concave hexagon. Sinusoidal curved edge honeycomb, (SCEH), tangential of ligamentous multi-ring stretching honeycomb (TMH), and link of ligaments multi-ring stretching honeycomb (LMH). The structure of the intraclass concave hexagonal cell is shown in Figure 1. In Figure 1, L is the width of the cell, H is the height of a single cell, t is the thickness of the cell, R_T_ is the radius of the ring in the cell of TMH, and R_L_ is the radius of the ring in the cell of LMH.

In the in-plane crushing of the honeycomb structure, the energy absorption characteristics in the deformation process are closely related to the relative density of the honeycomb structure. The relative density can be expressed as the ratio of the cross-sectional area of a single cell to the rectangular area of the cell contour. In Figure 1, the relative density of each cell is as follows:

(1) $${\bar{\rho }}_{RHH}=\frac{2Ht+2Lt\sin\alpha }{\sin\alpha }$$

(2) $${\bar{\rho }}_{TMH}=\frac{24\pi R_{T}Ht\sin\alpha +2Lt\sin\alpha -12R_{T}Ht\sin\alpha +8Ht}{HL\sin\alpha }$$

(3) $${\bar{\rho }}_{CCH}=\frac{16\pi -16\pi \cos(2\pi^{2}/H)+2HLt}{H^{2}L}$$

(4) $${\bar{\rho }}_{LMH}=\frac{24\pi R_{L}t\sin\alpha +2Lt\sin\alpha -16R_{L}t\sin\alpha +2Ht}{HL\sin\alpha }$$

### 2.2. Performance Evaluation of Cellular Structures

The mechanical properties of the honeycomb structure are mainly determined by the stress plateau and the energy absorption when the honeycomb structure is impacted [28]. The stress plateau of the honeycomb structure is expressed as:

(5) $$\sigma_{p}=\frac{1}{\varepsilon_{d}-\varepsilon_{y}}\int_{\varepsilon_{y}}^{\varepsilon_{d}}\sigma (\varepsilon )d\varepsilon$$

The index to evaluate the energy absorption capacity of honeycomb structures can be expressed in terms of specific energy absorption [28]. It represents the ability of the honeycomb structure to absorb energy per unit mass. The specific energy absorption of honeycomb structures can be expressed as:

(6) $$SEA=\frac{E}{m}=\frac{\int_{\varepsilon y}^{\varepsilon_{d}}\sigma (\varepsilon )d\varepsilon }{\bar{\rho }\rho_{s}}$$

where $\sigma_{p}$ is the platform stress of the honeycomb structure, $\varepsilon_{y}$ is the yield strain of the honeycomb structure, $\varepsilon_{d}$ is the compactification strain of the honeycomb structure, m is the overall mass of the honeycomb structure, $\bar{\rho }$ is the relative density of the honeycomb structure, $\rho_{s}$ is the density of the substrate that makes up the honeycomb structure, and *E* is the total energy absorbed.

### 2.3. Analysis of the Critical Velocity

According to the one-dimensional impact wave theory [29], the expression of the first critical velocity of honeycomb structures can be obtained as follows:

(7) $$V_{w}=\int_{0}^{\varepsilon_{cr}}\sqrt{\frac{d\sigma }{d\varepsilon }\frac{1}{\rho^{*}}}d\varepsilon$$

The expression for the second critical velocity is as follows:

(8) $$V_{s}=\sqrt{2\sigma_{p}\varepsilon_{d}/\rho^{*}}$$

where $\varepsilon_{cr}$ is the strain at the first strain peak when the honeycomb structure is subjected to an in-plane impact, $\rho^{*}$ can be expressed as $\rho^{*}=\bar{\rho }\rho_{s}$, and $\varepsilon_{d}$ is the strain at the beginning of the compaction phase when the honeycomb multi-empty plate is subjected to in-plane impact.

## 3. Finite Element Analysis

**Figure 2:**
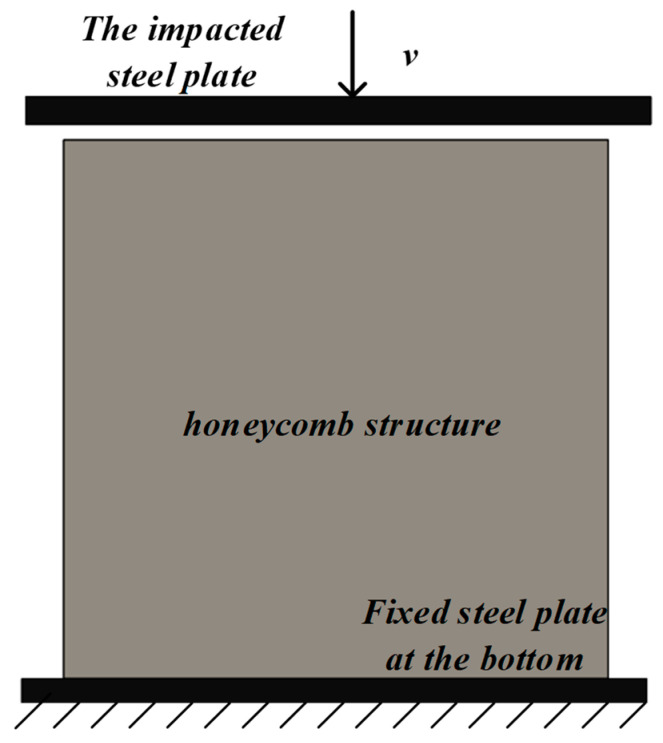
Diagrammatic sketch of impact mode.

**Figure 3:**
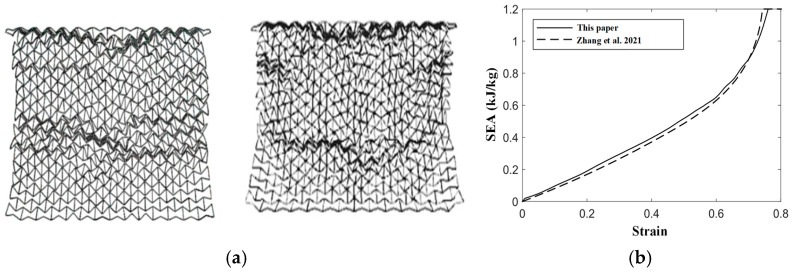
Finite element verification. (**a**) The deformation modes caused by the boundary conditions in this paper compared with those in the reference [30], (**b**) The comparison between the SEA caused by the boundary conditions in this paper and the SEA in the references [30].

In this paper, explicit dynamics is used for the finite element analysis. The structural matrix material mentioned in the paper is PLA. The matrix material is assumed to be an ideal elastoplastic material. The material parameters of PLA are as follows: Young’s modulus $E_{s}$ of 2360 MPa, yield stress $\sigma_{ys}$ of 33 MPa, the density ρ of $1.24\times 10^{3}\;{\mathrm{kg}/m}^{3}$. Shell elements are used in the finite element analysis process and the mesh is divided into S4R elements. In order to verify the reliability of the finite element model established in this paper, the same finite element model as in reference [30] is established: the honeycomb material is based on aluminum, and Young’s modulus $E_{s}$ of 69 GPa, yield stress $\sigma_{ys}$ of 76 MPa, the density ρ of $2.698\times 10^{3}\;{\mathrm{kg}/m}^{3}$ in the references, the Poisson’s ratio ν of 0.33. The out-of-plane thickness of the model is 1 mm and the arrangement of the cells in the honeycomb structure is 14×15. The friction between the honeycomb structure and the steel plate is regarded as smooth. The boundary conditions of finite element analysis are that the bottom steel plate is fixed, the top steel plate provides kinetic energy, and the *Z*-axis direction of the honeycomb structure is limited (as shown in Figure 2). The validation model of references is as follows (as shown in Figure 3): the angle is 15° and the impact velocity is 20 m/s.

### 3.1. Analysis of Impact Characteristics under Static Compression

The length and width of RHH, SCEH, TMH, and LMH are kept the same. The structural parameters of the four types of cells are as follows: H=30 mm, L=36 mm, α=50°, and t=1.2 mm. The thickness of the honeycomb structure is 10 mm. The ring radius of TMH and LMH are $R_{T}=R_{L}=2\;\mathrm{mm}$. To ensure stability during impact, the arrangement of the cells in the honeycomb structure is 6×30. The material parameters of PLA in ABAQUS are as follows: Young’s modulus $E_{s}$ of 2360 MPa, yield stress $\sigma_{ys}$ of 33 MPa, the density ρ of $1.24\times 10^{3}\;{\mathrm{kg}/m}^{3}$, and the Poisson’s ratio ν of 0.35. In order to ensure that the honeycomb structure is subjected to quasi-static impact, an ultra-low velocity of 1 m/s is used to simulate the impact in ABAQUS. The deformation patterns and performance parameters of honeycomb structures are observed under quasi-static impact. The differences between three kinds of concave hexagonal honeycomb structures and the traditional similar concave hexagonal honeycomb structures in the concave direction are analyzed in the quasi-static compression.

**Figure 4:**
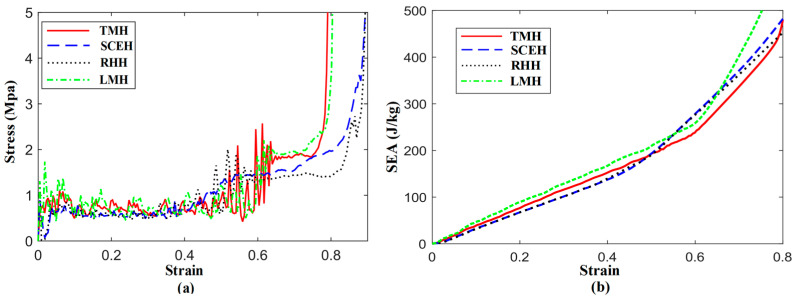
(**a**) Stress–strain curve of four honeycomb structures under 1 m/s, (**b**) SEA-strain curve of four honeycomb structures under 1 m/s.

In Figure 4a, when subjected to quasi-static impact in the concave direction, the four kinds of honeycomb structures show the phenomenon of a double stress plateau. The phenomenon does not occur when the other directions of the traditional concave hexagon are impacted. The first stress platform of TMH and LMH is higher than RHH and SCEH. LMH has the highest initial stress peak of the four honeycomb structures. TMH and LMH have a higher second stress plateau compared with SCEH and RHH. In Figure 4b, when the strain of the honeycomb structure is between 0 and 0.4, the energy absorption effect of SCEH and TMH is better than that of RHH and LMH under the same loading conditions. The energy absorption effect of KMH and RHH is almost the same. When the strain of the four honeycomb structures is greater than 0.5, LMH has the best energy absorption effect. In general, the in-plane performance of the similar concave hexagonal honeycomb structures is better than that of the traditional concave hexagonal honeycomb structures under quasi-static impact.

**Figure 5:**
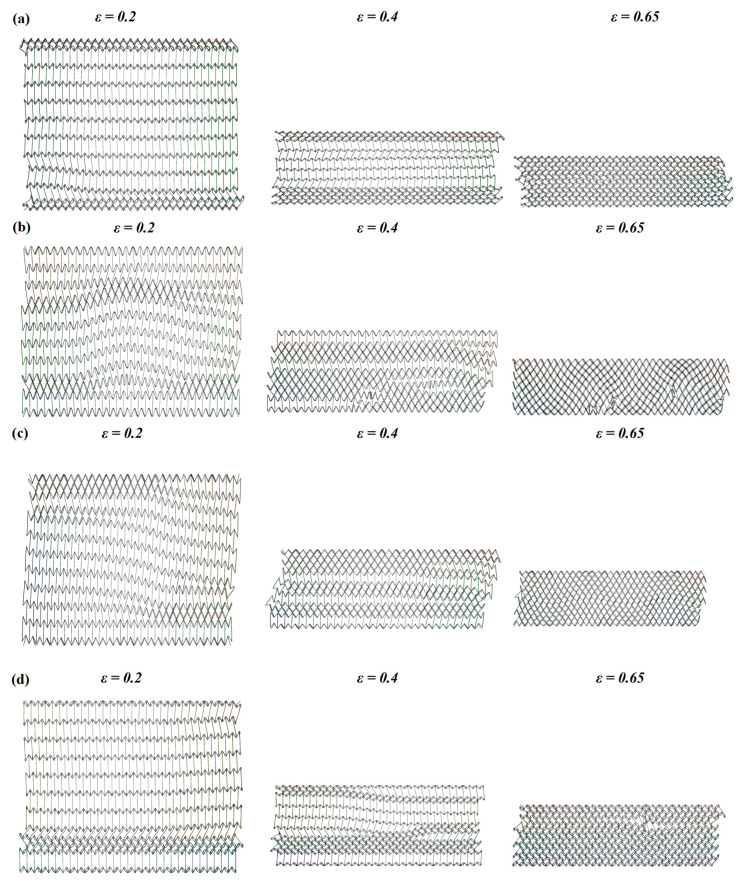
Deformation mode of the honeycomb structure under 1 m/s. (**a**) Deformation of TMH at different strains, (**b**) deformation of SCEH at different strains, (**c**) deformation of RHH at different strains, (**d**) deformation of LMH at different strains.

In terms of deformation mode, TMH and LMH are more stable than SCEH and RHH when subjected to in-plane impact, under the premise of the same cell size and number. As shown in Figure 5, when the strain of honeycomb structure reaches *ε* = 0.4, SCEH and RHH show greater skew compared with TMH and LMH.

In conclusion, through the comparison of four kinds of honeycomb structures in quasi-static impact, the in-plane performance of the similar concave hexagonal honeycomb structure is better than that of the traditional concave hexagonal honeycomb structure. In order to avoid repeated conclusions, three kinds of similar concave hexagonal honeycomb structures are analyzed in the following sections.

### 3.2. Analysis of Impact Characteristics of Similar Concave Hexagonal Honeycomb Structures under Different Impact Velocities

Based on the structure parameters in 3.1 and according to Equations (2)–(4), the relative density of the honeycomb structure composed of the three kinds of cells can be obtained as follows: ${\bar{\rho }}_{TMH}=0.151$, ${\bar{\rho }}_{LMH}=0.155$, ${\bar{\rho }}_{SCEH}=0.157$. According to the relative densities of the three types and Equations (7) and (8), the critical velocities of the three types of honeycomb structures can be obtained as shown in Table 1. According to the three types of critical velocity in the table, the low velocity impact velocity is 1 m/s, the medium velocity impact velocity is 30 m/s, and the high velocity impact velocity is 70 m/s. Since the low velocity of 1 m/s has been discussed in the previous section, only the impact characteristics under the medium velocity and high velocity conditions are discussed in this section.

#### 3.2.1. In-Plane Response at Different Impact Velocities

**Figure 6:**
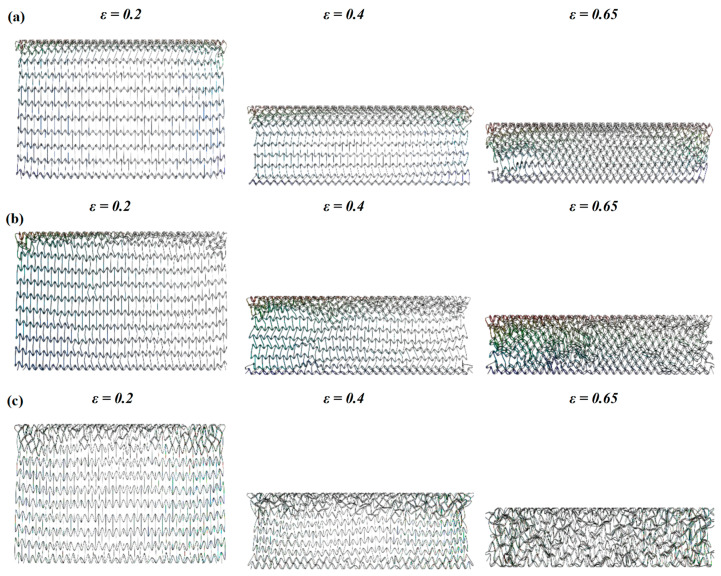
Deformation mode of honeycomb structure under 30 m/s. (**a**) Deformation of TMH at different strains, (**b**) deformation of LMH at different strains, (**c**) deformation of SCEH at different strains.

**Figure 7:**
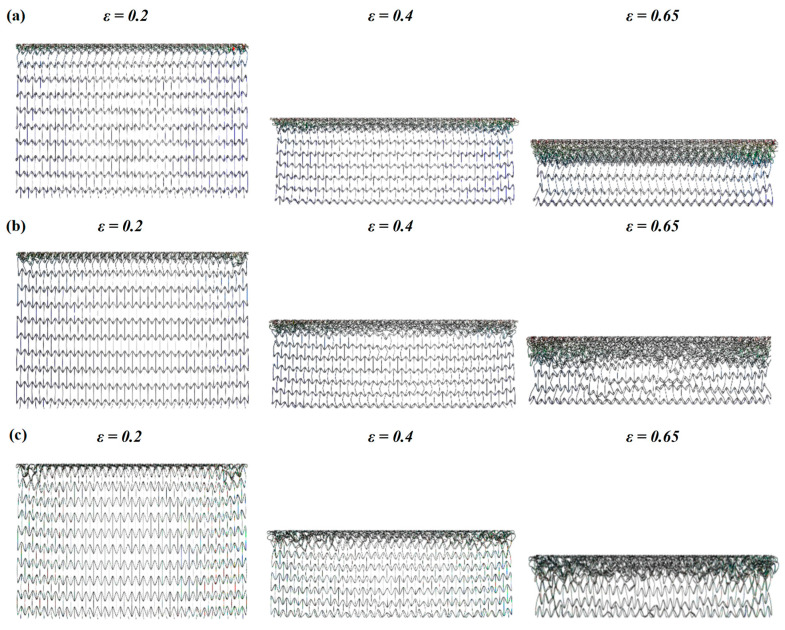
Deformation mode of honeycomb structure under 70 m/s. (**a**) Deformation of TMH at different strains, (**b**) deformation of LMH at different strains, (**c**) deformation of SCEH at different strains.

**Figure 8:**
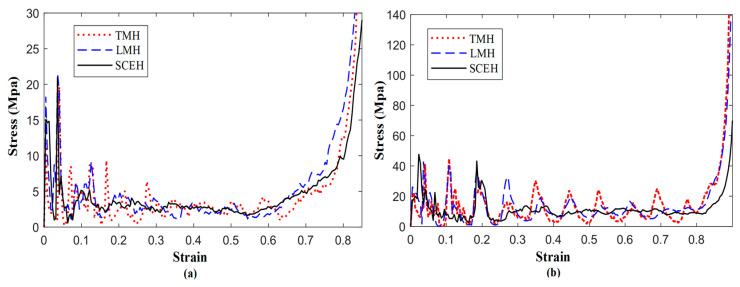
Stress–strain curve of three honeycomb structures at different velocities. (**a**) 30 m/s, (**b**) 70 m/s.

In Figure 6 and Figure 7, the deformation modes of the three honeycomb structures under medium and high velocity impact are shown. By combining the deformation modes in Section 3.1 we can obtain the following conclusions: there is no normal “V”-shape deformation band or “X”-shape deformation band [23] when the concave direction of the similar concave hexagonal honeycomb structure is impacted. When the honeycomb structure is impacted by the concave direction, a special “—” shape deformation band appears, and, with the increase in impact velocity, the first appearance of the “—” deformation band in the honeycomb structure appears to move up. With the fusion of the “—”-shaped deformation bands of each layer of the honeycomb structure, the second regular structure shape appears under low velocity impact. In the second shape of the honeycomb structure, the shape of a single cell is shown as a parallelogram. This is a sign of the appearance of a second stress platform. In the medium velocity impact, the honeycomb structure also produced a “—”-shaped deformation band, but mainly appeared in the top and low layers of the honeycomb structure, with the overall appearance of an “I” shape. When their strain reaches 0.65, LMH and SCEH are compressed into irregular shapes and TMH maintains the parallelogram shape. This leads to an unclear second stress plateau in TMH. In the high velocity impact, due to inertia the deformation of the honeycomb structure only occurs at the top. The overall deformation band of the “T” shape appears and the structure below the “—” shape deformation band is relatively stable. When the strain reaches 0.65, the regular quadrilateral structure does not appear, but the irregular structure does appear. In Figure 8a,b and the deformation mode diagram of the honeycomb structure, with the increase in the impact velocity, the compression deformation is mainly concentrated to the impact end because of inertia and the adhesive structure is formed between the cells. The parallelograms used for overloading do not appear, causing the second stress platform to blur or disappear.

**Figure 9:**
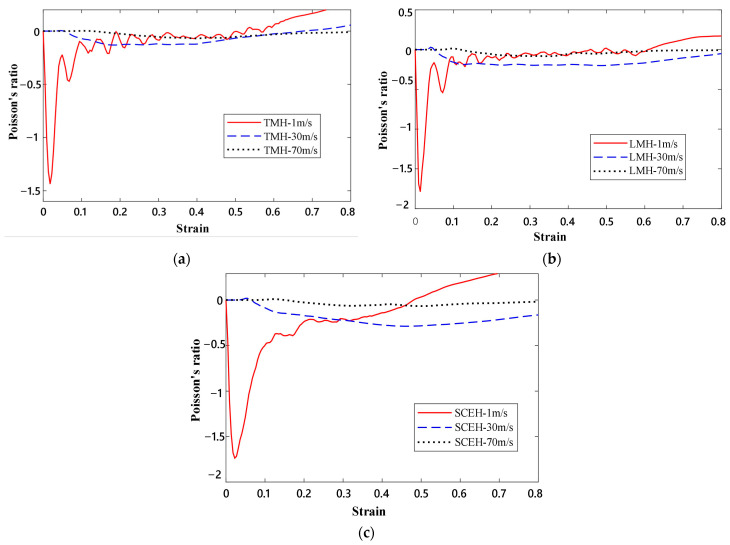
The Poisson’s ratio of three honeycomb cells at different impact velocities. (**a**) Poisson’s ratio of TMH at different velocities, (**b**) Poisson’s ratio of LMH at different velocities, (**c**) Poisson’s ratio of SCEH at different velocities.

In Figure 9, the dynamic Poisson’s ratios of the three honeycomb structures are shown when the concave direction of the three honeycomb structures is impacted at different impact velocities. The dynamic Poisson’s ratio of the honeycomb structure is more affected at low velocity impact and the dynamic Poisson’s ratio of the three honeycomb structures is smaller at low and medium velocities when they are impacted in the concave direction. With the increase in the impact velocity, the dynamic Poisson’s ratio in the concave direction of the honeycomb structure is less and less affected by the velocity. When the concave direction of honeycomb structure is impacted by high velocity, the dynamic Poisson’s ratio of the honeycomb structure is close to zero. With the increase in velocity, the difference in the dynamic Poisson’s ratio in the concave direction of the three honeycomb structures becomes smaller and smaller.

#### 3.2.2. Energy Absorption Characteristics at Different Impact Velocities

**Figure 10:**
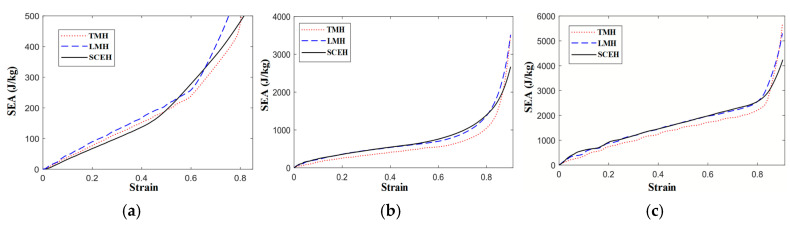
SEA-strain of three honeycomb structures at different velocities. (**a**) 1 m/s, (**b**) 30 m/s and (**c**) 70 m/s.

Based on Equation (6), the specific energy absorption curves of TMH, LMH, and SCEH structure cells under different impact velocities are obtained, as shown in Figure 10.

In Figure 10, when the impact is at low velocity, the specific energy absorption of TMH and LMH is better than that of SCEH, but when the three strains reach about 0.5, the energy absorption effect of the two cells is relatively weak. According to the deformation patterns of the three honeycomb structures at low velocities, the reason for the appearance of this structure is that SCEH entered the transition stage of parallel four-deformation earlier than TMH and LMH. When the three structures are subjected to medium velocity or high velocity impact, the inertial effect is enhanced and the relative density of TMH is minimal. As a result, the specific energy absorption of TMH is lower than that of the other two honeycomb structures at medium or high velocity impact. When the strain of the three honeycomb structures reaches 0.8, the honeycomb structure enters the compaction stage. Due to the unique ring structure of TMH and LMH, the specific energy absorption effect of TMH and LMH is much higher than that of SCEH after the strain reaches 0.8.

#### 3.2.3. Structural Deformation Analysis of a Single Cell

**Figure 11:**
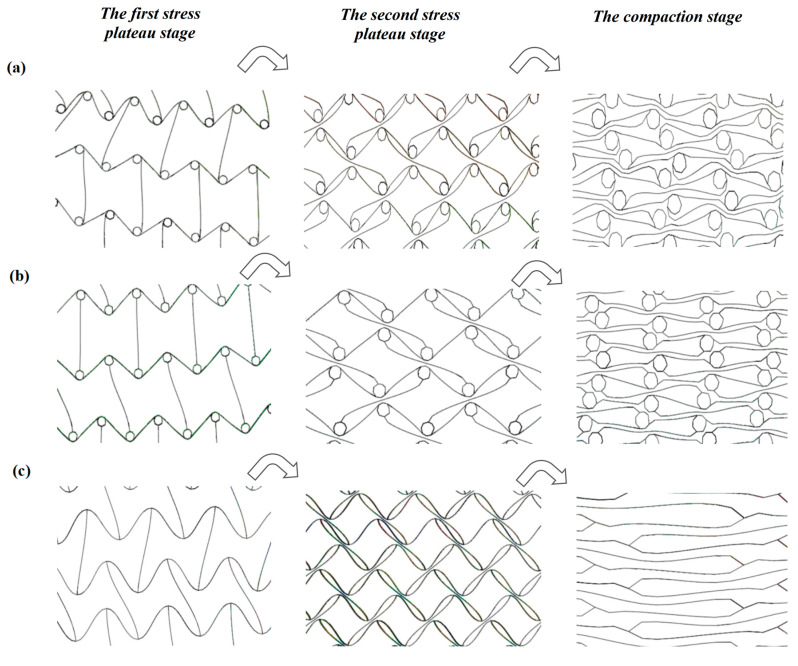
Deformation trend of unit cell of three honeycomb structures. (**a**) TMH; (**b**) LMH; (**c**) SCEH.

When the concave hexagonal honeycomb structure is impacted in the other direction, the unit cell only shows the concave contraction of the cell wall. However, when the concave direction of the concave hexagonal honeycomb structure is impacted, the unit cell in the honeycomb structure will collapse regularly. Finally, it will form a regular parallelogram structure (as shown in Figure 11). The special deformation mechanism causes the generation of the second stress plateau and the unusual change in the specific energy absorption at low velocity impact.

### 3.3. Influence of Structural Parameters

#### 3.3.1. The Effect of α in the In-Plane Impact

In order to research the influence of the α on the platform stress σ_p_ and the specific energy absorption of three honeycomb structures, the influence of different α on the stress plateau and the specific energy absorption of three honeycomb structures was studied when the cell wall was 1.2 mm and was impacted by 1 m/s.

**Figure 12:**
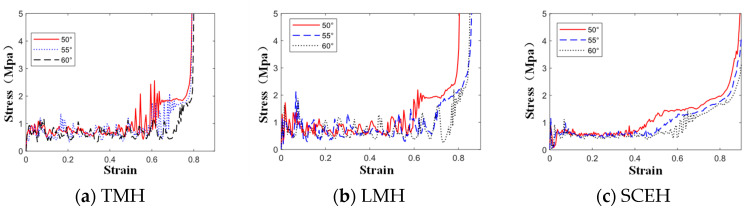
Stress–strain curve of three honeycomb structures with different α under 1 m/s.

**Figure 13:**
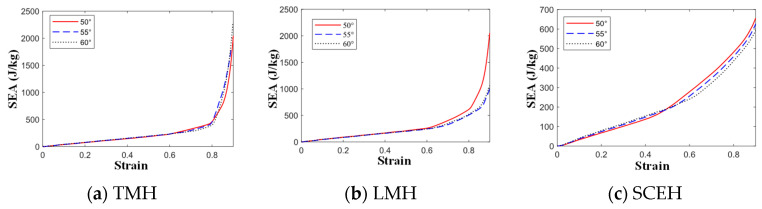
SEA-strain curve of three honeycomb structures with different α under 1 m/s.

As shown in Figure 12, the α is mainly the second stress plateau in the honeycomb structure. The second stress plateau in the three honeycombs becomes more ambiguous as the α decreases, and when α = 60, the second stress plateau in the three honeycomb structures becomes very ambiguous. When the α increases, the second stress platform will increase and extend, which means that the impact resistance is better. In Figure 13, It can be observed that changing α has little effect on the energy, but, when the strain value of LMH and SCEH is approximately 0.5–0.6, the smaller α will greatly improve the specific energy absorption of the honeycomb structure.

#### 3.3.2. The Effect of t in the In-Plane Impact

In order to research the influence of the *t* on the platform stress *σ_p_* and the specific energy absorption of three honeycomb structures, the influence of different *t* on the stress plateau and specific energy absorption of three honeycomb structures was studied when the α was 50° and was impacted by 1 m/s.

**Figure 14:**
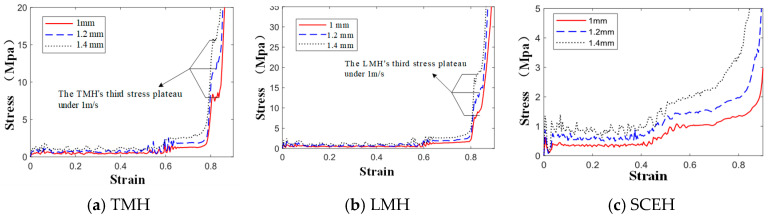
Stress–strain curve of three honeycomb structures with different t under 1 m/s.

**Figure 15:**
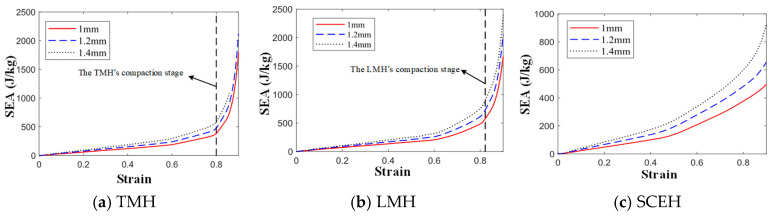
SEA-strain curve of three honeycomb structures with different t under 1 m/s.

According to the stress–strain curve in Figure 14, the *t* of the cell will simultaneously affect the first stress plateau, the second stress plateau, and the third stress plateau of the similar hexagonal honeycomb structure. The stress plateau will rise as the *t* increases. A short, third stress plateau will appear when the strain of TMH and LMH reaches 0.8. This phenomenon occurs because the ring in the cell plays an important role in the compaction stage of TMH and LMH. The following conclusions can be obtained by comparing Figure 13 and Figure 15: changing *t* will increase the specific energy absorption more than changing α, when the concave direction of the similar concave hexagonal honeycomb structure is impacted. When the strain of TMH and LMH reaches 0.8, the inflection point will appear in the value of specific energy absorption. The inflection point is because of the effect of the ring in the compaction stage of TMH and LMH.

## 4. Conclusions

In this paper, we compared the in-plane performance of similar concave hexagonal honeycomb structures when they are impacted by concave direction. TMH, LMH, and SCEH have a better in-plane performance than RHH in the concave direction. The in-plane response and specific energy absorption of three similar concave hexagonal honeycombs were analyzed in the concave direction. We arrived at the following conclusions:

(1) When a similar concave hexagon honeycomb structure is subjected to low-velocity impact in the concave direction, the honeycomb structure appears in two shapes. The two shapes are concave hexagonal morphology and parallel quadrilateral morphology. The first stress plateau corresponds to the concave hexagonal shape, and the second stress plateau is marked by the appearance of a parallelogram shape. In addition, TMH and LMH contain a ring structure, and a transient third stress plateau will appear when entering the compaction stage. The multi-platform mechanism improves the impact resistance of the honeycomb structure. It is beneficial to the application of multi-condition impact resistance.

(2) In the analysis of in-plane responses, TMH, LMH, and SCEH have a second stress platform similar to RHH under low-velocity impact. The stress plateau of the similar hexagonal honeycomb structure is higher than that of RHH and the phenomenon indicates that the three kinds of similar concave hexagonal honeycomb structures are better than RHH under low-velocity impact in the concave direction. In the medium velocity impact and high velocity impact, the inertia generated by the impact will end the cell glue phenomenon and the honeycomb structure cannot form a stable second shape. This causes the second stress platform to blur or even disappear. If the stress platform needs to be raised, it can be changed by α or t. The *t* affects the stress of all platforms in the concave direction of the similar hexagonal honeycomb structure and the α mainly affects the second stress plateau.

(3) In the analysis of specific energy absorption, under low velocity impact, the specific energy absorption of TMH and LMH is better than that of SCEH on the whole. The specific energy absorption of SCEH and LMH is better than that of TMH under medium and high velocity impact. SCEH will enter the second shape stage earlier than TMH and LMH, so when the strain of the honeycomb structure reaches about 0.5, the specific energy absorption of SCEH will be higher than that of TMH and LMH. If the stress platform needs to be raised, it can be changed by *t.* The α has little effect on the specific energy absorption of the honeycomb structure in the concave direction.

(4) In the analysis of the deformation modes of three kinds of honeycomb structures, the skew of SCEH is larger than TMH and LMH under low-velocity, medium-velocity, and high-velocity impact.

## Figures and Tables

**Table 1:** LMH, SCEH, and LMH critical velocity.

	TMH	SCEH	LMH
V_w_ (m/s)	1.77	1.44	1.2
V_s_ (m/s)	35	32	34

## Data Availability

This manuscript has associated data in a data repository. All data included in this manuscript are available upon request by contacting the corresponding author.

## References

[1] Lakes R. (1987). Foam Structures with a Negative Poisson’s Ratio. Science.

[2] Grima J.N., Evans K.E. (2000). Auxetic behavior from rotating squares. J. Mater. Sci. Lett..

[3] Lakes R. (1991). Deformation mechanisms in negative Poisson’s ratio materials: Structural aspects. J. Mater. Sci..

[4] Taylor M., Francesconi L., Gerendás M., Shanian A., Carson C., Bertoldi K. (2014). Low Porosity Metallic Periodic Structures with Negative Poisson’s Ratio. Adv. Mater..

[5] Zeng W., Ao Y., Yu S., Liu J., Liu J., Huang W. (2022). Investigation on the dynamic behavior of composite gradient double-arrow auxetic structure under local impact load. Polym. Compos..

[6] Almgren R.F. (1985). An isotropic three-dimensional structure with Poisson’s ratio = −1. J. Elast..

[7] Li J., Cui X.-Z., Qi H., Jin Q., Su J.-W., Wang Y.-L., Zhang X.-N. (2021). Laboratory tests on engineering properties of a new negative-Poisson′s-ratio geobelt. Constr. Build. Mater..

[8] Mardling P., Alderson A., Jordan-Mahy N., Le Maitre C.L. (2020). The use of auxetic materials in tissue engineering. Biomater. Sci..

[9] Budarapu P.R., Sudhir Sastry Y.B., Natarajan R. (2016). Design concepts of an aircraft wing: Composite and morphing airfoil with auxetic structures. Front. Struct. Civ. Eng..

[10] Alderson K., Alderson A., Anand S., Simkins V., Nazare S., Ravirala N. (2012). Auxetic warp knit textile structures. Phys. Status Solidi (b).

[11] Pan K., Ding J., Zhang W., Zhao S. (2021). The Negative Poisson’s Ratio Ship Base Design and Vibration Isolation Performance Analysis. Appl. Sci..

[12] Guo Y., Han X., Wang X., Fu Y., Xia R. (2021). Static cushioning energy absorption of paper composite sandwich structures with corrugation and honeycomb cores. J. Sandw. Struct. Mater..

[13] Toyoda M., Sakagami K., Takahashi D., Morimoto M. (2011). Effect of a honeycomb on the sound absorption characteristics of panel-type absorbers. Appl. Acoust..

[14] Lu T. (1999). Heat transfer efficiency of metal honeycombs. Int. J. Heat Mass Transf..

[15] Strek T.J., Matuszewska A., Jopek H. (2017). Finite Element Analysis of the Influence of the Covering Auxetic Layer of Plate on the Contact Pressure. Phys. Status Solidi (b).

[16] Mrozek A., Strek T. (2022). Numerical Analysis of Dynamic Properties of an Auxetic Structure with Rotating Squares with Holes. Materials.

[17] Novak N., Starčevič L., Vesenjak M., Ren Z. (2019). Blast response study of the sandwich composite panels with 3D chiral auxetic core. Compos. Struct..

[18] Duncan O., Alderson A., Allen T. (2021). Fabrication, characterization and analytical modeling of gradient auxetic closed cell foams. Smart Mater. Struct..

[19] Imbalzano G., Tran P., Ngo T.D., Lee P.V. (2016). A numerical study of auxetic composite panels under blast loadings. Compos. Struct..

[20] Sarvestani H.Y., Akbarzadeh A., Niknam H., Hermenean K. (2018). 3D printed architected polymeric sandwich panels: Energy absorption and structural performance. Compos. Struct..

[21] Imbalzano G., Linforth S., Ngo T.D., Lee P.V.S., Tran P. (2018). Blast resistance of auxetic and honeycomb sandwich panels: Comparisons and parametric designs. Compos. Struct..

[22] Günaydın K., Eren Z., Kazancı Z., Scarpa F., Grande A.M., Türkmen H.S. (2019). In-plane compression behavior of anti-tetrachiral and re-entrant lattices. Smart Mater. Struct..

[23] Liu W., Wang N., Luo T., Lin Z. (2016). In-plane dynamic crushing of re-entrant auxetic cellular structure. Mater. Des..

[24] Alomarah A., Masood S.H., Ruan D. (2022). Metamaterials with enhanced mechanical properties and tuneable Poisson’s ratio. Smart Mater. Struct..

[25] Li X., Lin Y., Lu F. (2019). Numerical Simulation on In-plane Deformation Characteristics of Lightweight Aluminum Honeycomb under Direct and Indirect Explosion. Materials.

[26] Alderson A., Alderson K.L., Chirima G.T., Ravirala N., Zied K. (2010). The in-plane linear elastic constants and out-of-plane bending of 3-coordinated ligament and cylinder-ligament honeycombs. Compos. Sci. Technol..

[27] Li Z., Gao Q., Yang S., Wang L., Tang J. (2017). Comparative study of the in-plane uniaxial and biaxial crushing of hexagonal, re-entrant, and mixed honeycombs. J. Sandw. Struct. Mater..

[28] Dong Z., Li Y., Zhao T., Wu W., Xiao D., Liang J. (2019). Experimental and numerical studies on the compressive mechanical properties of the metallic auxetic reentrant honeycomb. Mater. Des..

[29] Hönig A., Stronge W. (2002). In-plane dynamic crushing of honeycomb. Part I: Crush band initiation and wave trapping. Int. J. Mech. Sci..

[30] Zhang X.C., Liu Y., Li N. (2012). In-plane dynamic crushing of honeycombs with negative Poisson’s ratio effects. Explos. Shock Waves.

